# Boudin trafficking reveals the dynamic internalisation of specific septate junction components in *Drosophila*

**DOI:** 10.1371/journal.pone.0185897

**Published:** 2017-10-04

**Authors:** Camille Tempesta, Assia Hijazi, Bernard Moussian, Fernando Roch

**Affiliations:** 1 Centre de Biologie du Développement (CBD), Centre de Biologie Intégrative (CBI), Université de Toulouse, CNRS, UPS, Toulouse, France; 2 Lebanese University, Faculty of Sciences I and V—Doctorate School of Science and Technology-PRASE, Campus Rafic Hariri, Hadath-Beirut, Lebanon; 3 University of Tübingen, Interfaculty Institute of Cell Biology, Section Animal Genetics, Tübingen, Germany; Texas A&M International University, UNITED STATES

## Abstract

The maintenance of paracellular barriers in invertebrate epithelia depends on the integrity of specific cell adhesion structures known as septate junctions (SJ). Multiple studies in *Drosophila* have revealed that these junctions have a stereotyped architecture resulting from the association in the lateral membrane of a large number of components. However, little is known about the dynamic organisation adopted by these multi-protein complexes in living tissues. We have used live imaging techniques to show that the Ly6 protein Boudin is a component of these adhesion junctions and can diffuse systemically to associate with the SJ of distant cells. We also observe that this protein and the claudin Kune-kune are endocytosed in epidermal cells during embryogenesis. Our data reveal that the SJ contain a set of components exhibiting a high membrane turnover, a feature that could contribute in a tissue-specific manner to the morphogenetic plasticity of these adhesion structures.

## Introduction

The septate junctions (SJ) are the cell adhesion structures accounting for the maintenance of selective paracellular barriers in invertebrate tissues [1]. They play thus an analogous role to that of vertebrate tight junctions, as both prevent the unrestricted diffusion of macromolecules, ions and solutes through epithelial layers and are essential for the physiological compartmentalisation of internal organs [2]. Despite their different structural organisations, these two types of occluding junctions display significant parallelisms at the molecular level, as they are known to include different homologous components [3].

The SJ owe their name to a stereotyped organisation characterised by the presence of regularly spaced bridges, known as septa, which span the intercellular space and connect the lateral membranes of adjacent cells [4]. The molecular characterisation of different *Drosophila* mutants exhibiting defective barriers has permitted the identification in this insect of a large number of SJ structural components [5]. These include different membrane proteins, like the claudins Megatrachea (Mega), Kune-kune (Kune) and Sinuous (Sinu) [6–8], the adhesion molecules NeurexinIV (NrxIV), Neuroglian (Nrg) and Contactin (Cont) and the α and β subunits of the Na^+^/K^+^ ATPase (ATPα and Nrv2) [9–12]. In addition, the SJ contain several cytoplasmic adaptors such as Coracle (Cor), Varicose (Vari) and Yurt, which establish links with the cortical cytoskeleton [13–15]. Multiple developmental studies have revealed that the SJ constituents are extremely interdependent for their clustering in the membrane sub-apical region, suggesting that the integrity of these adhesion structures relies on the preservation of a highly organised molecular complex [5]. Moreover, studies in live embryos have shown that a set of SJ core components integrated by Nrg, NrxIV, the two Na^+^/K^+^ ATPase subunits and the adaptor Cor forms a stable structure at the membrane level [16,17]. However, how the maintenance of stable molecular assemblies is compatible with the cell rearrangements observed in developing epithelia is not known.

In this report we present evidence indicating that two specific *Drosophila* SJ components are internalised during epithelial morphogenesis and associate to SJ in a dynamic way. These two membrane proteins, Kune [8] and Boudin (Bou) [18] are respectively members of the claudin and Ly6 gene families, both including multiple paralogs necessary for SJ formation [5]. Kune is one of the *Drosophila* homologues of the claudin family, which were first characterised as tight junction components [8]. In vertebrates, the extracellular loops of these proteins can establish contacts with the claudins of contiguous cells and are thus directly implicated in the formation of paracellular barriers [19]. However, the role of their insect counterparts remains enigmatic, as it is thought that they cannot exert an equivalent function in the SJ due to the large gap separating adjacent membranes in invertebrate epithelia [20]. Several *Drosophila* Ly6 proteins are known as well to be required for SJ integrity, but their specific molecular functions have not been fully characterised [18,21–23]. It has been proposed that these small GPI-anchored membrane ligands [18,22] could play an indirect role in SJ formation, contributing to the intracellular trafficking of other SJ components [23]. However, it is not clear whether the Ly6 proteins participate in the membrane routing of specific SJ components or in other aspects of intracellular traffic, such as their endocytic recycling [23]

Using live imaging techniques, we have shown that Bou is a SJ component displaying a unique property, as it can travel extracellularly and integrate into the junctions of distant cells. In addition, our observations indicate that this protein is internalised in membrane vesicles containing another SJ constituent, the protein Kune. Our results reveal that, similarly to its vertebrate homologues, this claudin is endocytosed at the SJ level in epidermal cells, suggesting that these adhesion structures include dynamically regulated components that could contribute to junctional plasticity during epithelial morphogenesis.

## Results

### Boudin is an essential SJ component

**Fig 1:**
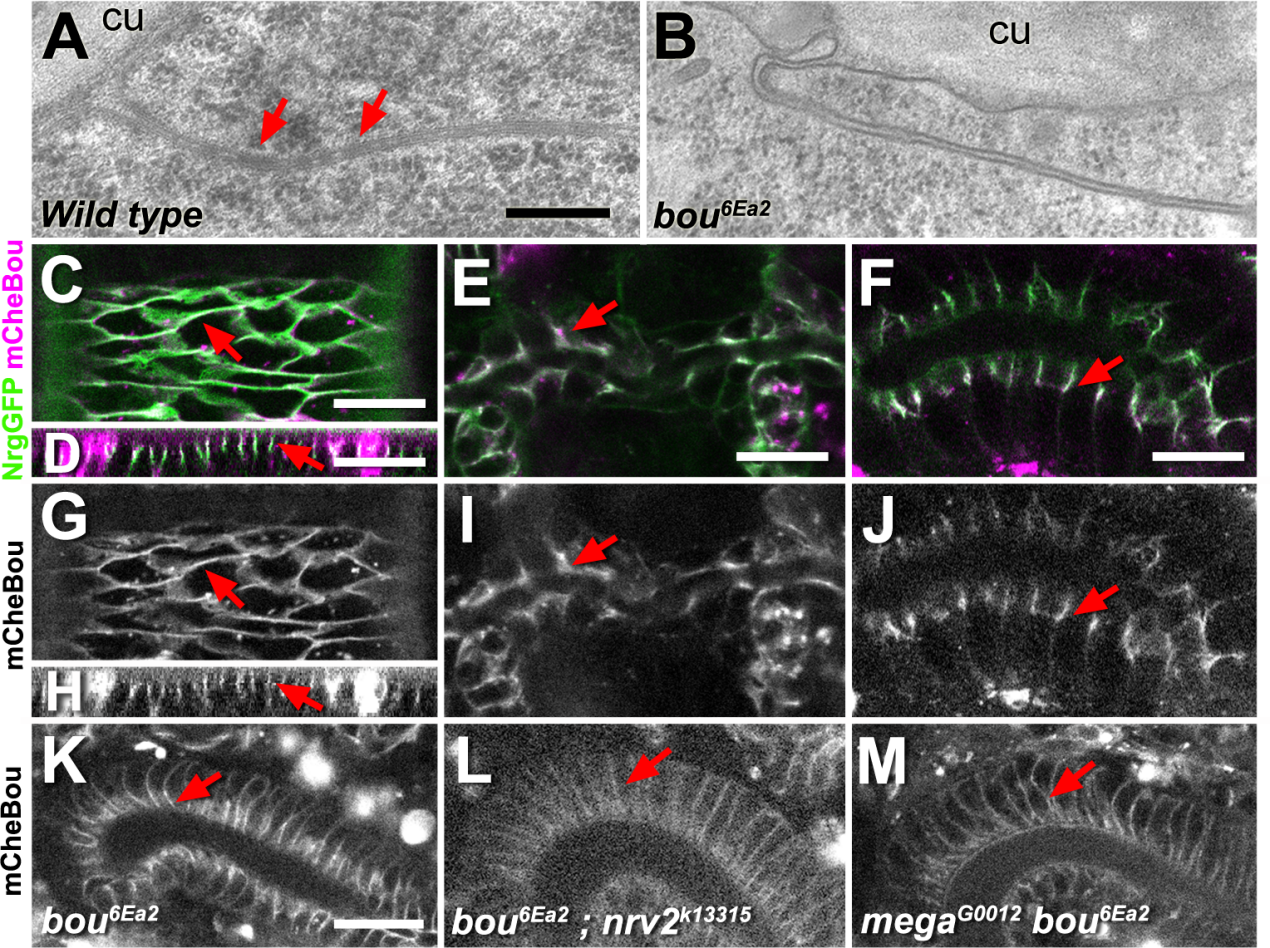
Bou is a SJ structural component. (A,B) Transmitted electron micrographs showing the lateral membrane of contiguous epidermal cells in both wild type (A) and *bou* (B) stage 17 embryos. An electron dense material corresponding to septa is found in the intercellular space of contiguous wild type cells (A, red arrows). These structures are completely absent in the cell contacts of *bou* embryos. The epidermal cuticle is also indicated (cu). Scale bar: 25 nm. (C-J) Confocal images corresponding to stage 16 live *bou* embryos expressing mCheBou under the control of *HhGAL4* in the epidermis (C,G: planar view, D,H: transverse view), dorsal tracheal trunk (E,I) and salivary gland (F,J). The mCheBou protein (magenta in C-F, b/w in G-J) accumulates at the level of the SJ (red arrows), co-labelled with Nrg-GFP (green). Scale bars: 15 μm. (K-M) Confocal images of live stage 16 embryos showing the distribution of mCheBou in the hindgut columnar epithelium of *bou* rescued embryos (K) or double mutants *bou; nrv2* (L) and *mega bou* (M). In the double mutants the mCheBou membrane signal is uniformly distributed over the lateral membrane, instead of accumulating in its most apical part (red arrows). Scale bar: 20 μm.

Previous analysis has shown that the Ly6 protein Boudin (Bou) is required during *Drosophila* development for paracellular barrier integrity and the clustering of SJ components in the membrane sub-apical region [18]. We have extended previous *bou* phenotypical characterisations by comparing the ultrastructure of cell adhesion junctions in the epidermis of stage 17 wild type and mutant embryos, using transmission electron microscopy. Whereas in control embryos we observe the presence of an electron-dense material bridging the lateral cell membranes of contiguous cells (Fig 1A), these structures are totally absent in the cell contacts of *bou*^*6Ea2*^ null mutants (Fig 1B). Therefore, *bou* activity is necessary for the formation of septa, as it has been previously reported in many mutants exhibiting defects in SJ organisation and paracellular barrier integrity [6,10,24].

Former attempts to characterise the subcellular localisation of Bou relied on the observation in fixed samples of an epitope tagged version of this protein, revealing its capacity to travel from cell to cell [18]. Here, we studied the localisation of Bou in live embryos using a new mCherry-tagged form of Bou (mCheBou). In rescue experiments, expression of mCheBou in the *Hedgehog GAL4* (*HhGAL4*) domain is sufficient to elicit the emergence of adult hemizygotes for the *bou*^*6Ea2*^ null allele, indicating that the Bou tagged form retains its biological activity. We then analysed mCheBou localisation in live *bou* embryos expressing a SJ marker, the Nrg-GFP protein [25]. Driven by *HhGAL4*, mCheBou is detected in the epidermis by stage 12 and its levels gradually increase throughout development (S1 Fig). In all stages examined the protein diffuses away from its source and is incorporated by distant cells at the membrane level, where it co-localises with Nrg-GFP (Fig 1C and 1D and S1 Fig). Thorough examination of stage 16 rescued embryos reveals that mCheBou is also incorporated into the SJ of other ectodermal tissues, such as the tracheal dorsal trunk and the salivary glands (Fig 1E and 1F). These observations thus indicate that Bou could act as a structural component of these adhesion junctions. Moreover, instead of accumulating in the cell sub-apical region, mCheBou appears uniformly distributed over the lateral membrane in the columnar hindgut cells of *bou* rescued embryos that are also mutant for other SJ components, such as the Na^+^/K^+^ ATPase subunit *nrv2* or the claudin *mega* [6,11] (Fig 1K–1M). Therefore, mCheBou membrane clustering depends on SJ integrity, as it has been observed for most SJ structural components [5].

### Bou can travel extracellularly

**Fig 2:**
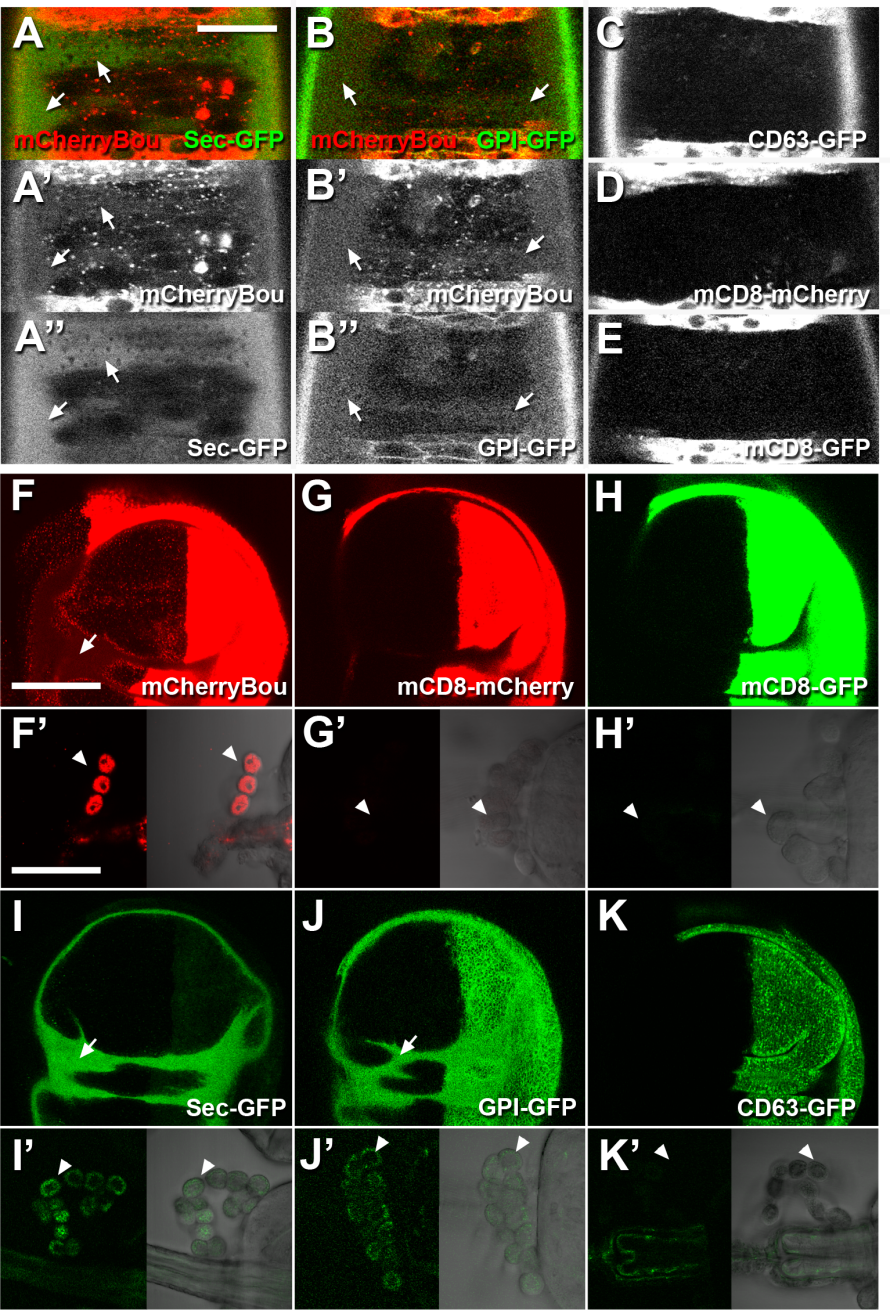
mCheBou diffuses systemically during embryonic and larval stages. (A-E) Confocal images showing the ventral epidermis of live stage 16 embryos expressing different fluorescent proteins in the *HhGAL4* domain, which is visible in segmental stripes. An extracellular diffuse signal corresponding to mCheBou (red in A,B, b/w in A’,B’) is detected in the perivitelline space (arrows), which also accumulates Secreted-GFP (green in A, b/w in A”) and GPI-GFP (green in B, b/w in B”, arrows). Neither mCD8mCherry (D) nor mCD8GFP (E) or CD63-GFP (C) are detected outside the *HhGAL4* stripes. Scale bar: 20 μm. (F-K,F’-K’) Confocal pictures corresponding to third instar larvae live explants of wing imaginal discs (F-K) and garland cells (F’-K’, visible in the bright field right panels). The different fusion proteins are produced in the *HhGAL4* domain, visible in the posterior compartment of the wing disc (F-K). mCherryBou (F,F’), Secreted-GFP (I,I’) and GPI-GFP (J,J’) are detected in the garland cells (white arrowheads) and in the extracellular space comprised between the disc peripodial membrane and the wing pouch (arrows). No signal could be detected in these locations upon expression of mCD8mCherry (G,G’), mCD8GFP (H,H’) or CD63-GFP (K,K’). Scale bars: 100 μm.

Our observations in live embryos show that mCheBou is secreted extracellularly, as we observe a diffuse mCherry signal in the space comprised between the vitelline membrane and the embryonic epidermis when this protein is expressed in the *HhGAL4* domain (Fig 2A–2B’ and S1 Fig). This extracellular mCheBou signal could correspond to a soluble form of this protein, as a secreted form of GFP (Sec-GFP) [26], is also found in the same location (Fig 2A and 2A”). In contrast, no extracellular signal could be detected upon expression of transmembrane forms of mCherry or GFP (Fig 2D and 2E). Previous studies in cultured cells have shown that a significant fraction of Bou is bound to the cell membrane by a GPI-anchor [18]. Surprisingly, we observe that a GPI-GFP form [27] also localises to the perivitelline space (Fig 2B and 2B”). Thus, a common mechanism could permit extracellular diffusion of different GPI-anchored proteins. One possibility is that they could travel associated to extracellular lipid vesicles, such as exosomes [28], but we did not detect a comparable extracellular signal when we expressed the CD63-GFP exosome marker [29] (Fig 2C). Thus, it is unlikely that exosomes could be a vector for mCheBou diffusion.

We also noticed that mCheBou diffuses into the body cavity of third instar larvae. In fact, upon expression with *HhGAL4*, mCheBou accumulates in the garland cells, a larval nephrocytic organ filtering the insect hemolymph [30] (Fig 2F’). Consistently with our observations in the embryo, garland cells also retain secreted fractions of GPI-GFP and Sec-GFP (Fig 2J’–2K’). In contrast, transmembrane mCD8-coupled forms of mCherry or GFP and the CD63-GFP exosome marker were not detected in these cells (Fig 2G’, 2H’ and 2K’), even if they are distinctly perceived in the *HhGAL4* posterior compartment of the wing imaginal discs (Fig 2G, 2H and 2K). In this tissue we also observe a specific fluorescent signal upon expression of mCheBou, Sec-GFP and GPI-GFP in the extracellular space confined between the peripodial membrane and the anterior part of the wing pouch (Fig 2F, 2I and 2J). Thus, our observations show that mCheBou is secreted extracelullarly and can diffuse systemically in both embryonic and larval stages.

### Nrg and Kune are required for Bou membrane localisation

**Fig 3:**
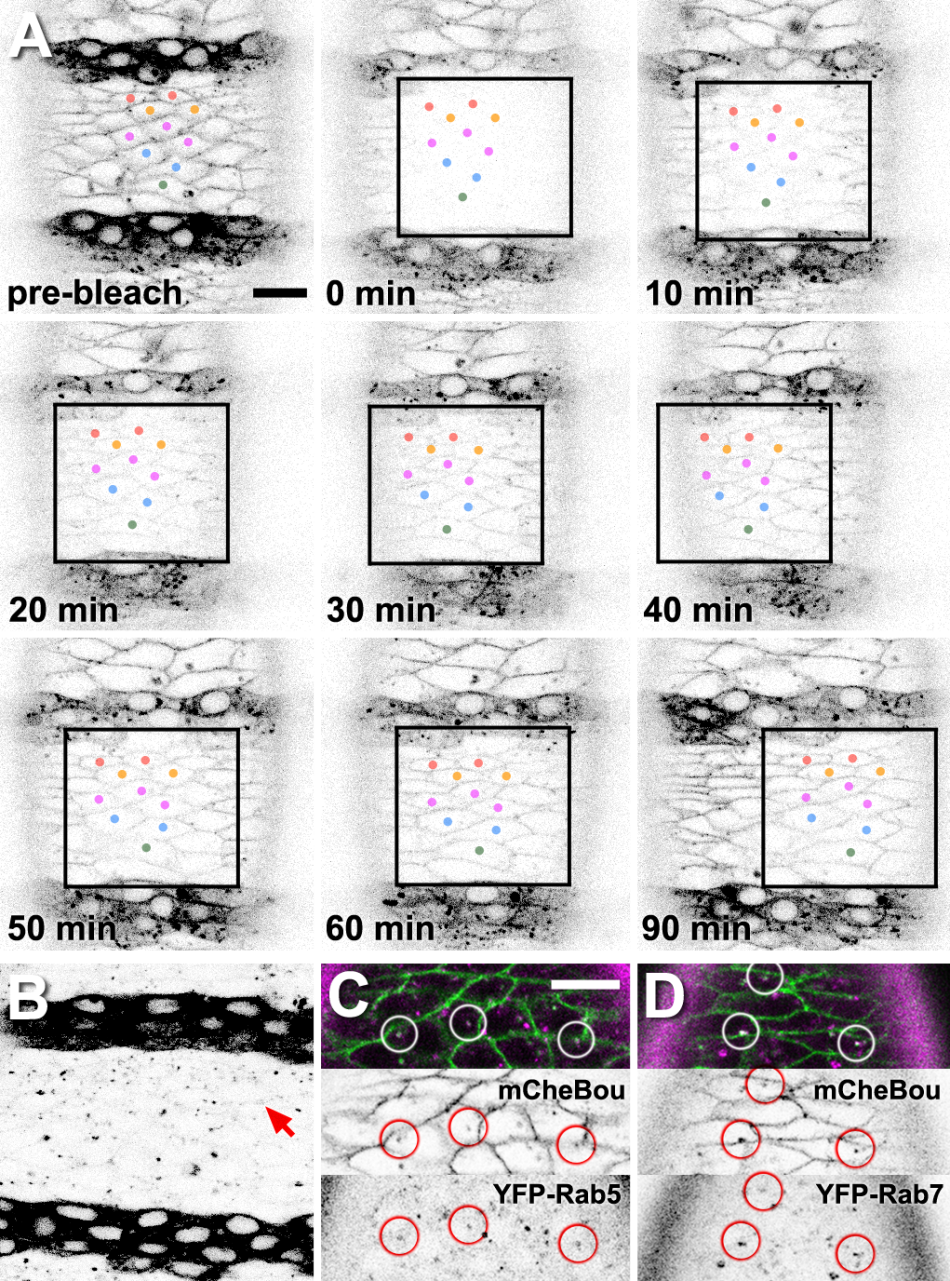
Exogenous mCheBou is captured and endocytosed in the embryonic epidermis. (A) Confocal optical sections showing the distribution of mCheBou in the ventral epidermis of a live stage 14 *bou* rescued embryo. After photobleaching (time 0) of an area delimited by a black square, the mCheBou signal gradually reappears in the cell cortex. Cells entirely bleached at time 0 are labelled with coloured dots, to facilitate their visualisation. (B) Confocal image showing the mCheBou distribution in the ventral epidermis of a live stage 14 wild type embryo, imaged using the same parameters as the rescued embryos shown in A. Only a faint signal is seen in the cell contours (red arrow). (C,D) mCheBou distribution (green in top panels, b/w in middle panels) in the ventral epidermis of live stage 14 *bou* rescued embryos expressing ubiquitously the early (YFP-Rab5, C) or late (YFP-Rab7, D) endosome markers (shown in magenta in top panels and on b/w, bottom panels). Examples of vesicles positive for mCheBou and each marker are labelled with circles. Scale bars: 10 μm.

To gain further insights into the process of exogenous Bou incorporation, we photo-bleached the mCheBou signal in epidermal cells not producing this protein and monitored fluorescence recovery at different intervals. Our observations reveal that the mCheBou levels recover gradually at the membrane level in stage 14 *bou* rescued embryos (Fig 3A), suggesting that the capture of exogenous mCheBou molecules is a dynamic process that could depend on the availability of a specific membrane recognition complex. Interestingly, this recognition capacity seems to be impaired in the presence of an endogenous Bou product, as the mCheBou signal is barely detected at the membrane in wild type embryos (Fig 3B).

Our analysis also reveals the presence of exogenous mChebou in intracellular puncta in both rescued and wild type epidermal cells (Fig 3B and 3D). To characterise these structures, we have studied mCheBou co-localisation with YFP-Rab5 and YFP-Rab7, two vesicular markers labelling respectively early and late endosomes [31]. In *bou* stage 14 rescued embryos we detect mCheBou in vesicles containing either marker, indicating that Bou can also be internalised by endocytosis and reach late endosomal compartments (Fig 3C and 3D).

**Fig 4:**
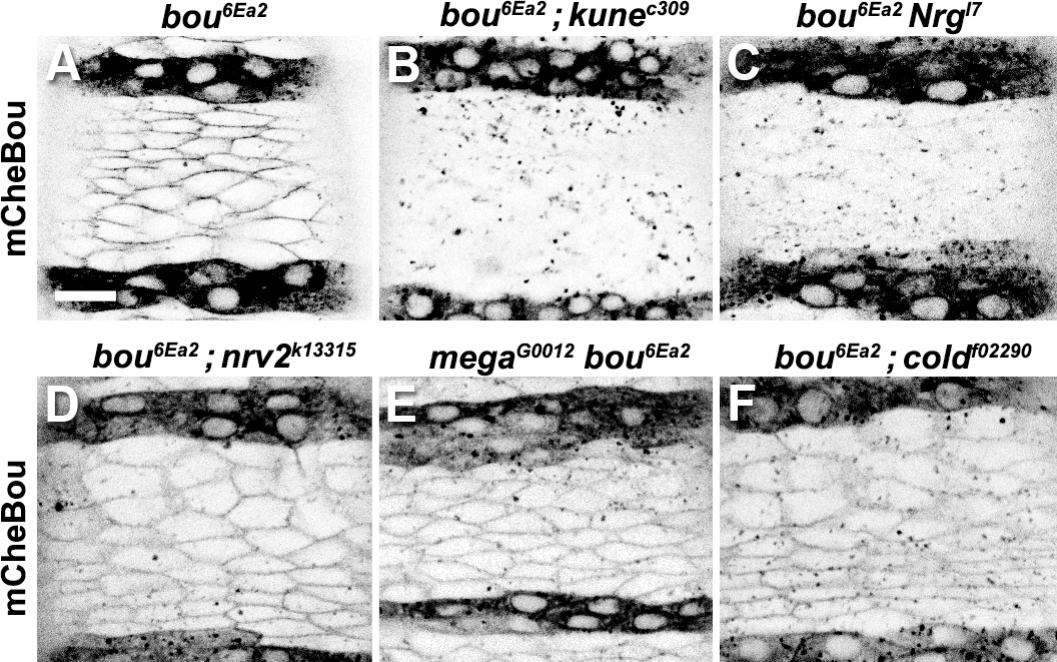
Membrane accumulation of exogenous mCheBou is impaired in specific SJ mutants. (A-F) Each panel shows a confocal image illustrating the localisation of mCheBou in the ventral epidermis of live stage 14 embryos of different genotypes, labelled accordingly. mCheBou is produced in the *HhGAL4* domain, visible in the dark epidermal stripes. A mCheBou membrane associated signal is detected in the cells of *bou* rescued mutants (A) and, at lower levels, in *bou; nrv2* (D), *mega bou* (E) and *bou; cold* (F) double mutants. No membrane mCheBou signal is detected in *bou; kune* (B) or *bou Nrg* (C) double mutant embryos, but the exogenous protein is seen in intracellular puncta. Scale bar: 10 μm.

We reasoned that other SJ components could be implicated in the recognition and/or internalisation of exogenous mCheBou, acting as receptors for this protein. We thus analysed exogenous mCheBou incorporation in live *bou* rescued embryos mutant for other SJ components. The genetic backgrounds tested included embryonic lethal alleles for two different claudins, *mega* and *kune*, the Ly6 protein *coiled* (*cold*), the adhesion protein *Nrg* and the Na^+^/K^+^ ATPase subunit *nrv2* [9,11,21]. We noticed that the exogenous mCheBou is readily detected in the cell membrane of *mega bou*, *bou cold* and *bou nrv2* double mutant embryos (Fig 4D–4F). In contrast, no membrane signal could be observed in either *bou kune* or *bou Nrg* epidermal cells (Fig 4B and 4C). Yet, mCheBou is seen in intracellular vesicles in these two genetic contexts, indicating that its secretion is not compromised and the mutant cells can incorporate this exogenous molecule. Therefore, whereas SJ integrity is not a prerequisite for extracellular mCheBou capture, the *Nrg* and *kune* products differ from the other SJ components analysed and could play a specific role in mCheBou membrane accumulation.

### Nrg is essential for Kune membrane localisation

**Fig 5:**
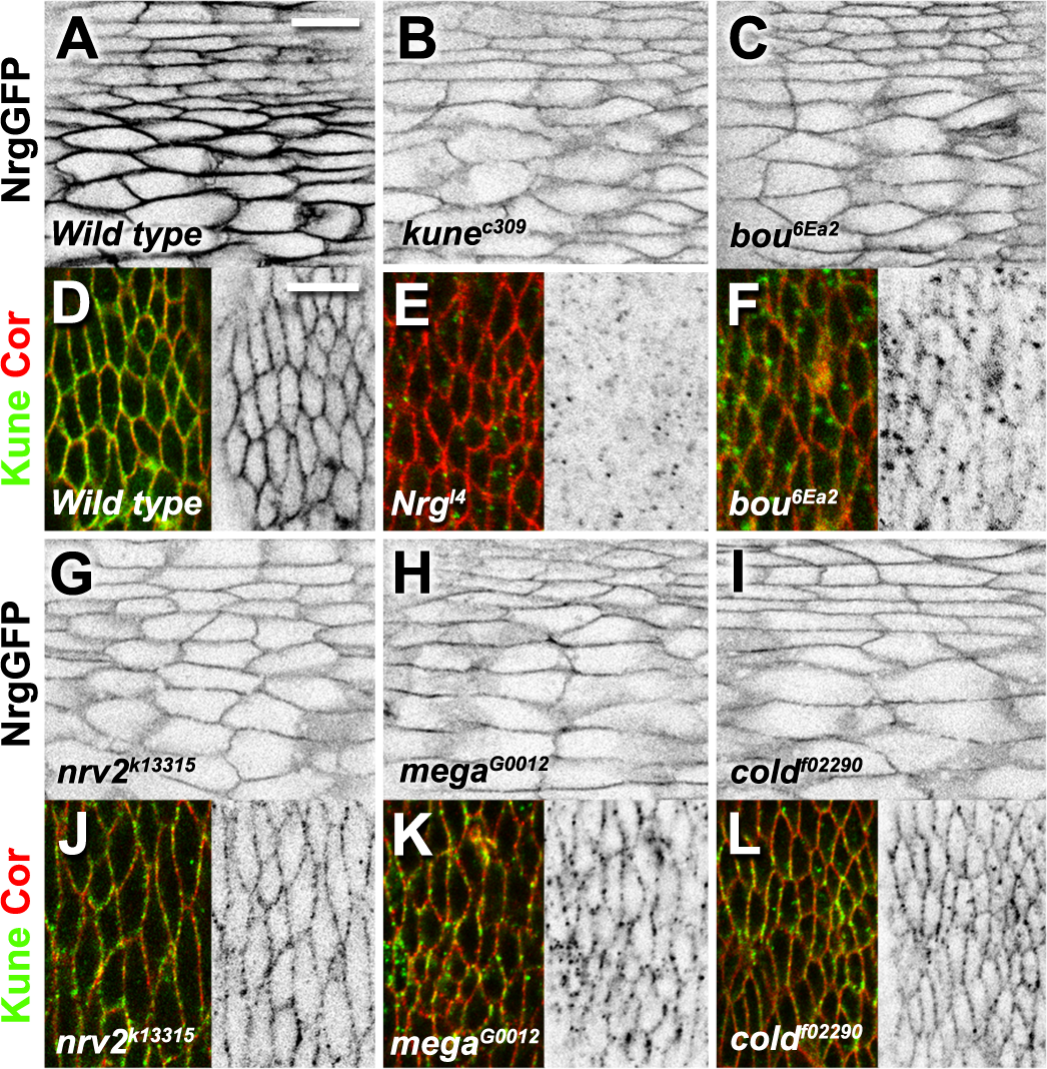
Kune localisation is specifically affected in *bou* and *Nrg* mutants. (A-C,G-I) Confocal images showing the localisation of Nrg-GFP in the ventral epidermis of live stage 16 embryos. In *kune* (B), *bou* (C), *nrv2* (G), *mega* (H) and *cold* (I) mutants Nrg-GFP membrane levels are lower than in wild type controls (A). (D-F,J-L) Confocal images showing the lateral epidermis of stage 16 embryos immunostained for Kune (green in left panels, b/w in right panels) and Coracle (red, left panels). Kune is detected in the cell membrane of *nrv2* (J), *mega* (K) and *cold* (L) mutant embryos, although at lower levels than in wild type controls (D). No Kune membrane staining is seen in *Nrg* embryos (E) and only a faint signal is detected in the cell contours of *bou* mutants (F). Scale bars: 10 μm.

To investigate specific interactions between *Nrg*, *kune* and *bou*, we compared the subcellular distributions of Kune and Nrg in mutants lacking the other two SJ components, and also in *mega*, *cold* and *nrv2* backgrounds. We find in the epidermis of stage 16 live embryos that Nrg-GFP localises to the membrane in all the mutants examined, although the intensity of the signal is reduced in comparison to wild type controls (Fig 5A–5C and 5G–5I). The Kune distribution was instead monitored with a specific antibody (Nelson et al., 2010) and compared to that of the Coracle SJ marker [13] (Fig 5D–5F and 5J–5L). The Kune membrane levels appear reduced to variable extents in the different SJ mutants tested, although in the *Nrg* embryos virtually no staining could be detected at the cell contours (Fig 5D–5F and 5J–5L). We also observe some variability in the Kune membrane levels in mutants for additional SJ components, including *NrxIV*, *Cont*, the claudin *sinu* and the Ly6 protein *crooked* (*crok*) [7,10,12,23] (S2 Fig). However, a significant fraction of Kune is always seen at the cell cortex in these backgrounds (S2 Fig). Thus, these observations reveal that *Nrg* plays a prominent role in the trafficking of Kune.

One possibility is that Kune entry into the protein secretory pathway could be blocked in absence of Nrg or other SJ components. A quality control mechanism such as the unfolded protein response (UPR) could detect an abnormal accumulation of Kune in the endoplasmic reticulum (ER) and trigger its subsequent degradation [32]. We thus evaluated the activation of the UPR pathway in embryos mutant for different SJ components, monitoring by RT-PCR the levels of a specific *Xbox binding protein 1* (*Xbp1*) splicing variant forming in ER stress conditions [33]. Our results show that the *Xbp1* unconventional splicing is clearly enhanced in *mummy* (*mmy*) mutants, where protein glycosylation and trafficking are severely disrupted [34], but is not up-regulated in *mega*, *bou* or *Nrg* null backgrounds (S2 Fig). Therefore, mutations affecting the SJ organisation do not necessarily activate a robust UPR response.

### Kune is endocytosed during epithelial morphogenesis

To gain insight into the dynamics of Kune trafficking, we decided to analyse its localisation in live embryos. For this, we engineered genomic constructs driving expression of two N-terminal EGFP and mCherry tagged forms of this claudin (S3 Fig). One copy of either construct is sufficient to rescue the SJ defects observed in homozygous embryos for the *kune*^*C309*^ null allele [8], indicating that the resulting tagged proteins have a normal activity (S3 Fig).

In the epidermis of live embryos expressing simultaneously both forms we observe already by stage 11 an EGFPKune signal associated to the cell membrane, with levels increasing in this location until the end of embryogenesis (S4 Fig). In contrast, the mCheKune fluorescence becomes visible at stage 12 and its cortical levels remain low until stage 17 (S4 Fig). Puzzlingly, immunostaining of the same embryos with specific antibodies against EGFP and mCherry reveals that the two Kune tagged forms co-localise at the membrane in all the stages examined (S4 Fig). These observations can however be reconciled acknowledging that in live cells newly synthesised EGFP matures faster and becomes fluorescent earlier than mCherry, which is in turn more resistant to the acidic environment of late endosomal compartments [35,36]. Thus, in live tissues most of the Kune protein could be internalised at the membrane level before the mCherry fluorescence becomes visible, as it has been previously shown for other *Drosophila* membrane proteins [37].

**Fig 6:**
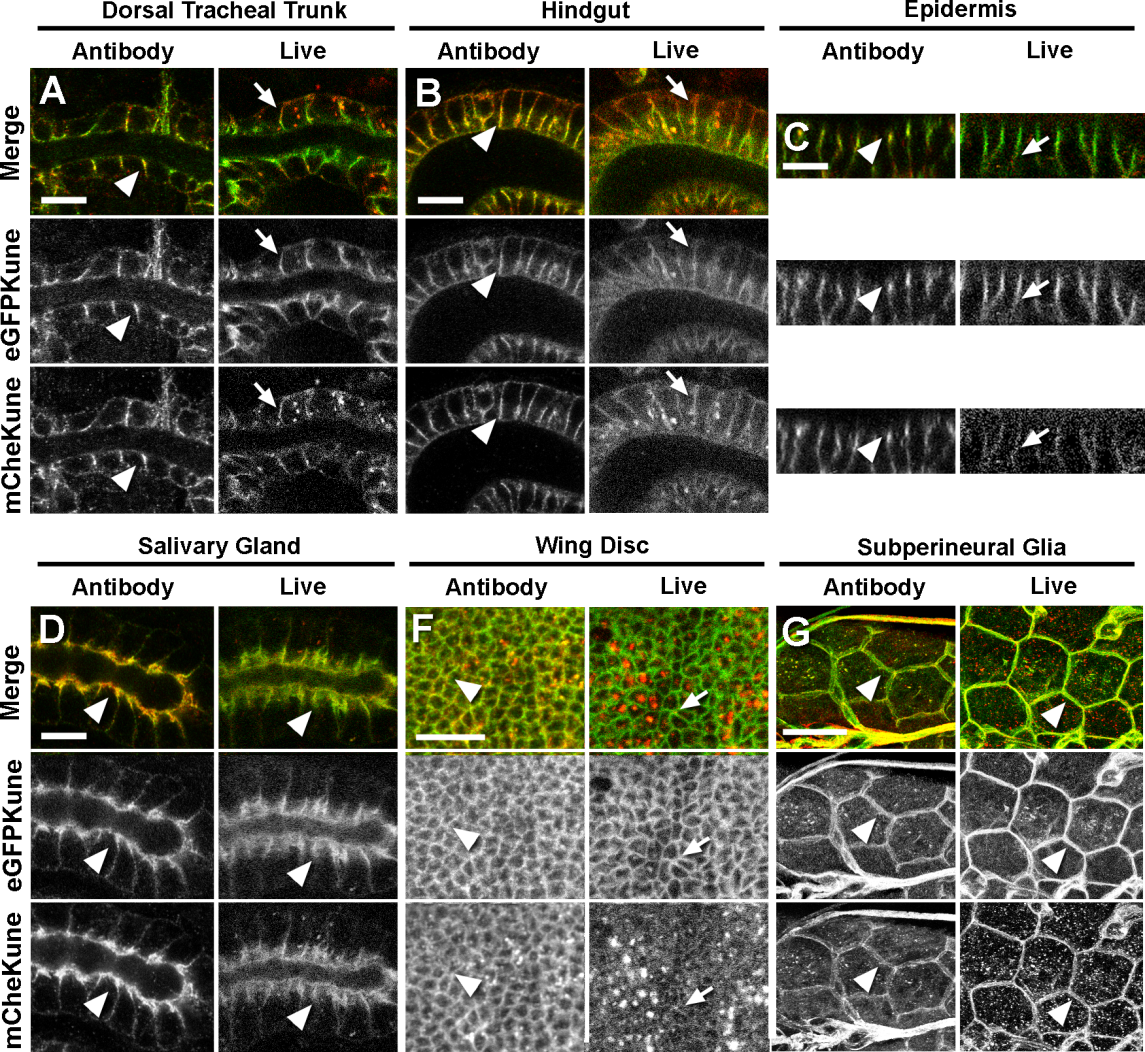
Live imaging reveals Kune membrane turnover in different cellular contexts. (A-G) Confocal optical sections corresponding to stage 16 embryos (A-D) and third larval instar tissues (F,G) expressing simultaneously EGFPKune and mCheKune. The left panels (Antibody) correspond to immunostainings with specific antibodies against GFP (green and b/w in middle panels) and mCherry (red and b/w in bottom panels). The right panels (Live) present the EGFPKune and mCheKune fluorescent signals observed in live samples. In immunostainings, the signals of the two tagged proteins coincide at the SJ level (arrowheads) in the embryonic tracheal dorsal trunk (A), hindgut (B), epidermis (C), salivary glands (D), the wing imaginal discs (F) and the larval subperineural glia (G). In live samples, a weak mCheKune signal is seen along the lateral membrane in all tissues (arrows), except in the salivary glands and the subperineural glia, where a strong signal coincides with EGFPKune at the SJ level (arrowheads). Scale bars: 10 μm (A-F) or 45 μm (G).

As in the epidermis, we observe in the tracheal cells and the hindgut epithelium of stage 16 embryos identical localisations for both proteins in immunostainings but different distributions of their fluorescent signals in live tissues (Fig 6A–6C). We noted that instead of accumulating in the sub-apical part of the cell, as the EGFPKune signal, the mCheKune fluorescence appears uniformly distributed over the baso-lateral membrane in these epithelia (Fig 6A–6C). In contrast, in the salivary glands we observe similar distributions for the EGFP and mCherry signals in both live and immunostained embryos (Fig 6D). Therefore, Kune membrane turnover could proceed at a slower rate in this specific cell context. We also analysed larval tissues known to form SJ, such as the wing disc epithelial cells [38] and the brain subperineural glia [39]. In wing disc live explants, mCheKune is barely detected at the SJ level, although its presence in this compartment is confirmed in immunostained samples (Fig 6F). In contrast, mCheKune is detected in the membrane of subperineural glial cells in both live and fixed tissues (Fig 6G). These examples thus suggest that in some tissues Kune could be associated with the SJ region in a more stable fashion.

**Fig 7:**
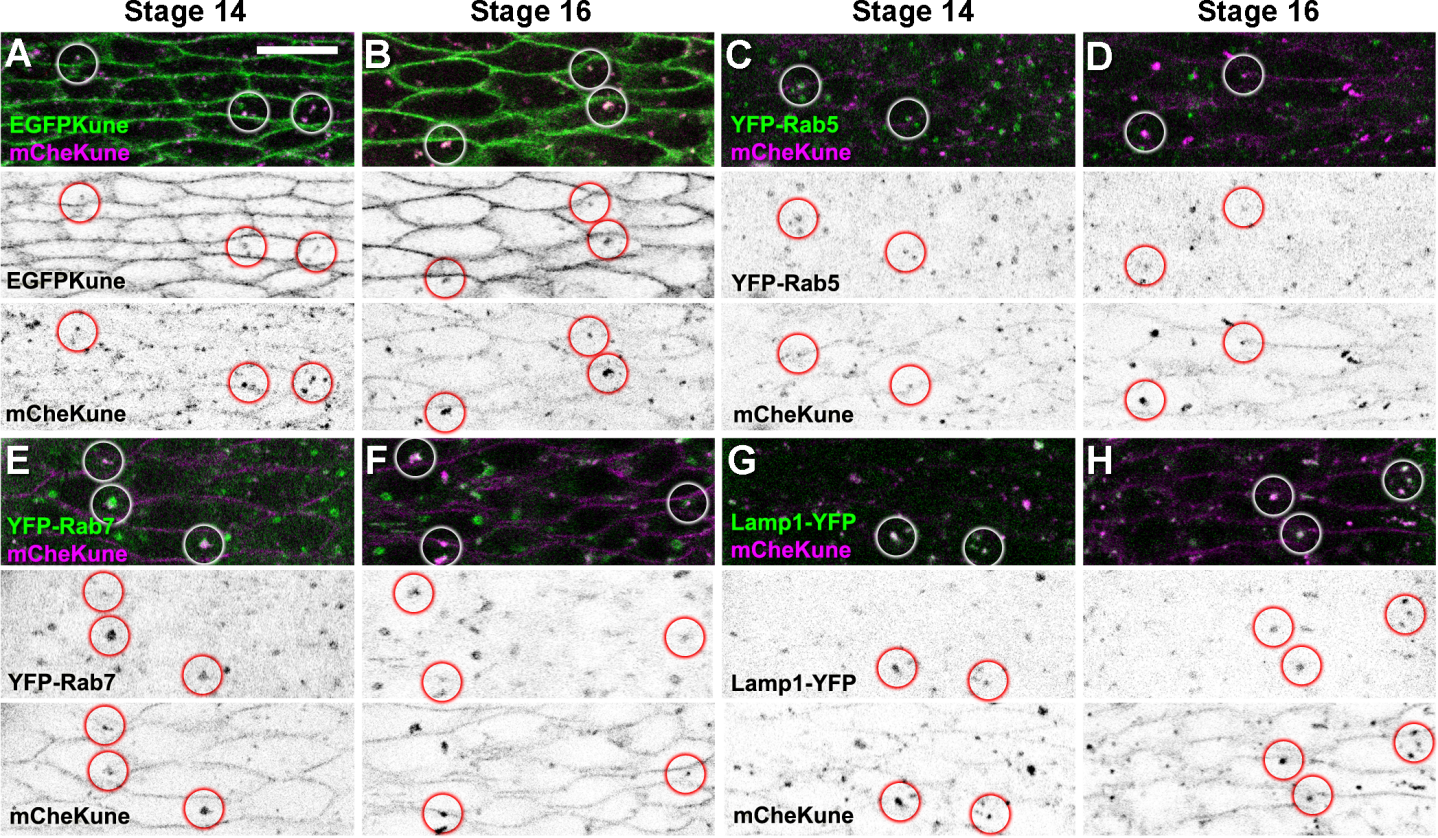
Kune is endocytosed and degraded during epidermal morphogenesis. (A-H) Confocal views of the ventral epidermis corresponding to live wild type stage 14 (A,C,E,G) or stage 16 (B,D,F,H) embryos expressing mCheKune (magenta and b/w in bottom panels) and EGFPKune (A,B), YFP-Rab5 (C,D), YFP-Rab7 (E,F) or Lamp1-YFP (G,H), all shown in green (top panels) and in b/w (middle panels). Vesicles containing both tagged Kune versions (labelled with circles) are visible in both stages (A,B). mCheKune is also detected in YFP-Rab5 early endosomes (C,D), YFP-Rab7 late endosomes (E,F) and Lamp-1-YFP lysosomes (G,H). Scale bar: 10 μm.

Supporting the idea that Kune is internalised in the embryonic epidermis, we detect throughout development the presence of intracellular puncta showing both mCherry and EGFP fluorescence, albeit the levels of green fluorescence are variable and often weak in these structures (Fig 7A and 7B and S4 Fig). Moreover, in both stage 14 and 16 live embryos we observe that mCheKune is present in vesicles labelled with YFP-Rab5, YFP-Rab7 or Lamp1-YFP (Fig 7C–7H), which respectively mark early endosomes, late endosomes and lysosomes [31,40]. Therefore, a fraction of the Kune protein is endocytosed and targeted for degradation during epithelial morphogenesis.

### Kune membrane turnover is enhanced in *Nrg* mutants

**Fig 8:**
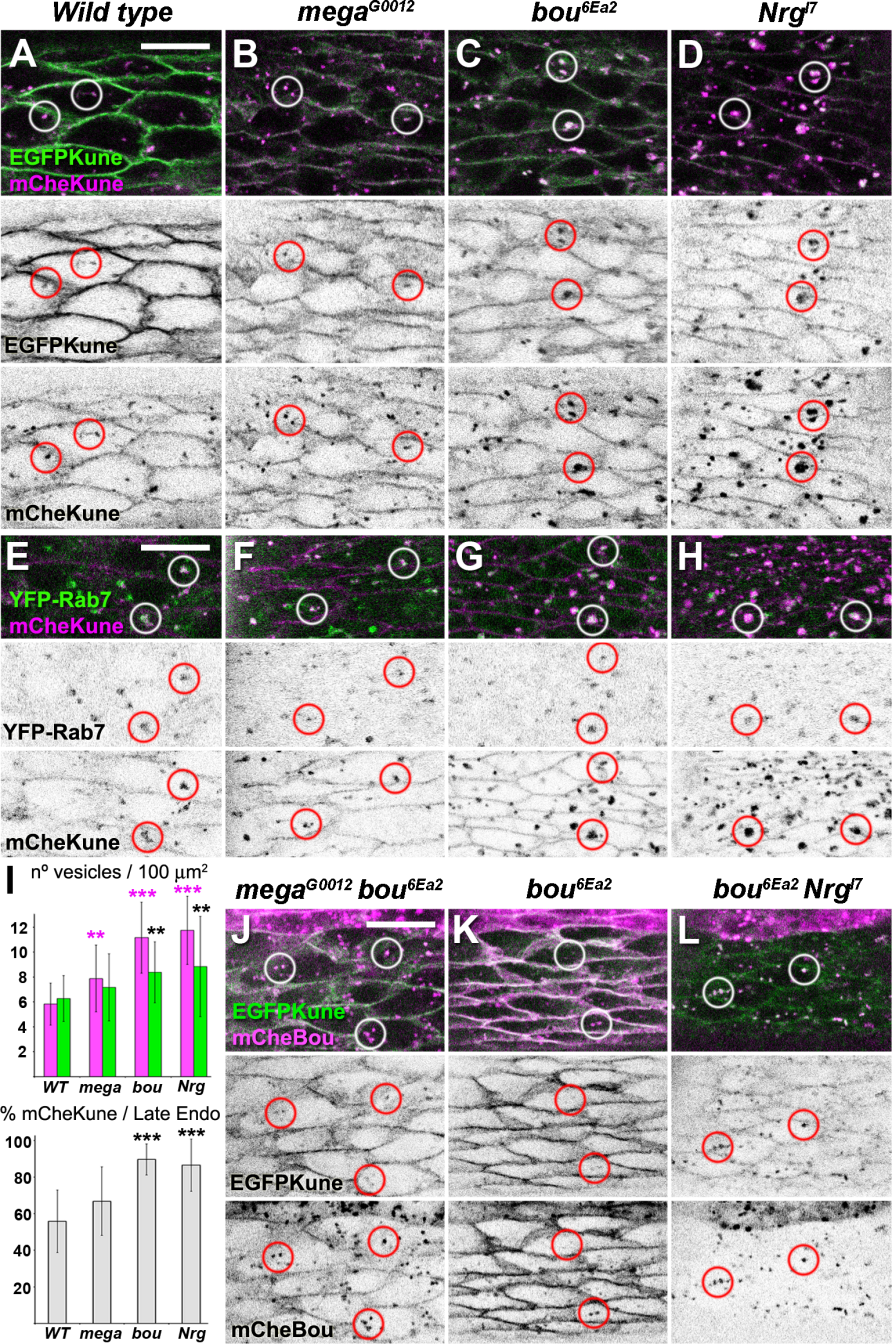
Kune membrane localisation is affected in SJ mutants. (A-H) Confocal images corresponding to the ventral epidermis of live stage 16 embryos expressing mCheKune (magenta and b/w in bottom panels) and EGFPKune (A-D, shown in green and b/w in middle panels) or YFP-Rab7 (E-H, shown green and b/w in middle panels). All images were acquired using the same parameters; specific genotypes are indicated above each column. The EGFPKune membrane levels are diminished with respect to wild type controls (A) in *mega* (B), *bou* (C) and especially in *Nrg* mutants (D). A weak mCheKune membrane signal is seen in all these backgrounds. mCheKune is also detected in internal vesicles containing EGFPKune (A-D) and YFP-Rab7 (E-H). These vesicles (labelled with circles) are more abundant in the *bou* (C and G) and *Nrg* (D and H) mutant embryos. (I) The upper graph represents for each genotype the average number of mCheKune (magenta) and YFP-Rab7 vesicles (green) found in regions of 100 μm ^2^ corresponding to the epidermis of stage 16 embryos. The bottom graph represents the average percentage of YFP-Rab7 late endosomes also positive for mCheKune. Error bars represent the standard deviations observed after pooling the data of different sample areas (n≥13). Both *bou* and *Nrg* mutants have more mCheKune and YFP-Rab7 vesicles (respectively, p<0.01 and p<0.05, Student’s t-test) and show a higher proportion of late endosomes containing mCheKune (p<0.01, Student’s t-test). (J-L) Ventral epidermis of stage 16 live embryos expressing EGFPKune (green, b/w in middle panels) and mCheBou (magenta, b/w in lower panels) under the control of *HhGAL4*. Imaging parameters are identical for all genotypes, indicated above each column. All the vesicles containing EGFPKune are also positive for exogenous mCheBou (examples are marked with circles). Scale bars: 10 μm.

We took advantage of the EGFP and mCherry forms to study Kune trafficking in conditions where the SJ organisation is compromised. For this, we analysed the localisation of these two versions in the epidermis of live stage 16 *mega*, *bou*, and *Nrg* mutant embryos. In comparison to wild type controls, the membrane levels of EGFPKune fluorescence are significantly reduced in all the mutants examined, especially in *Nrg* embryos (Fig 8A–8D and S5 Fig). In contrast, we detect in the same location a weak but comparable mCheKune signal in all the backgrounds tested (Fig 8A–8D and S5 Fig). We also noticed the presence of conspicuous mCheKune puncta displaying EGFPKune fluorescence, especially in *bou* and *Nrg* mutants (Fig 8A–8D). To characterise and quantify these structures, we counted the number of mCheKune vesicles present in wild type and mutant epidermal cells expressing the YFP-Rab7 late endosome marker (Fig 8E–8H). Our observations reveal an increase in the cellular pools of mCheKune and YFP-Rab7 vesicles in both *bou* and *Nrg* mutants (Fig 8I). In addition, the proportion of late endosomes also positive for mCheKune is augmented in these two mutant backgrounds (Fig 8I). Thus, an increasing fraction of Kune is sent to late endosomal compartments when SJ organisation is perturbed, although this effect is more pronounced in *Nrg* and *bou* embryos (Fig 8E–8I). We also examined if exogenously provided mCheBou is incorporated into the Kune vesicles. For this, we monitored mCheBou and EGFPKune localisation in *bou* rescued embryos and in *mega bou* and *bou Nrg* double mutants (Fig 8J–8L). We observe in these backgrounds that all the EGFPKune vesicles are positive for mCheBou, suggesting that these two proteins could be internalised together (Fig 8J–8L). Moreover, in *bou Nrg* embryos little EGFPKune and no mCheBou are observed at the membrane level (Fig 8L), thus confirming that *Nrg* plays a prominent role preventing the internalisation of these two SJ components.

## Discussion

### Bou is a septate junction structural component

Previous results revealed that four members of the extended *Drosophila* Ly6 family, Bou, Crim, Cold and Crok, are required in a non-redundant manner for the maintenance of the paracellular barrier and the organisation of SJ [18,21–23]. However, the molecular roles of these proteins have not been elucidated. Whereas it has been shown that Crim localises to the SJ [41], Bou, Crok and Cold have been detected in intracellular membrane compartments, suggesting that they could play an indirect role during SJ assembly [18,21–23]. We show in this report that Bou is essential for the formation of septa and behaves as a SJ component, localising to the plasma membrane and clustering at the level of these adhesion structures in different ectodermal tissues. Moreover, Bou diffuses baso-laterally in mutant cells for other SJ components, indicating that its membrane clustering depends on SJ integrity. Both live imaging and rescue experiments have been instrumental for evaluation of the Bou subcellular localisation, suggesting that immunostaining procedures previously used to characterise its distribution are too aggressive to preserve its association with the SJ. Our findings thus illustrate the advantages of non-invasive imaging techniques to study the assembly of these complex adhesion structures.

Our observations provide an explanation for the specific capacity of Bou to act in a cell non-autonomous manner [18], as we have shown that this protein can diffuse systemically and integrate into the SJ of distant cells. Whether other members of the *Drosophila* Ly6 family are also secreted is not known, but *cold*, *crim* and *crok* are thought to operate in a cell-autonomous way [21,23]. Therefore, it is possible that Bou could have specific features permitting its secretion. As predicted for Ly6 proteins, Bou has been found attached to the plasma membrane of cultured cells thanks to a GPI-anchor [18]. Thus, although exosomes are unlikely to be a vector for Bou extracellular diffusion, this protein could travel with an intact GPI-anchor associating with other lipid extracellular particles. Alternatively, as it is the case for some vertebrate GPI-anchored proteins, different endogenous activities could cleave the Bou GPI-moiety and release a soluble version of this protein [42]. Interestingly, upon expression in the fat body of a GPI-anchored form of mCherry, a soluble fraction of this protein can be recovered in the larval hemolymph [43]. Thus, *Drosophila* could encode for enzymatic activities capable of cleaving specifically the GPI-anchors of other proteins such as Bou or GPI-GFP, which can diffuse systemically.

Presently we can only speculate about the nature of the mechanisms managing the incorporation of exogenous Bou into functional SJ. For instance, a direct exchange between extracellular and SJ associated fractions could explain the assimilation of mCheBou into the junctions of *bou* rescued embryos, as observed in our photo-bleaching experiments. Interestingly, in wild type embryos we detect little membrane incorporation of mCheBou, indicating that presence of the endogenous product could hinder the capture of exogenous molecules. One possibility is that the endogenous Bou could be more stably associated to the cell membrane, perhaps due to its GPI-anchor, and block the integration of external molecules. However, although we have shown that the mCheBou version used in our analysis is functional, we cannot formally exclude that its tag could impair some of its natural capacities. In addition, more complex mechanisms involving rapid endocytosis and/or other intermediate steps could explain the available observations. Nevertheless, our data indicate that upon cell capture the exogenous Bou molecules associate at the membrane level with other SJ components. Consistently, we have shown that this protein clusters in the cell sub-apical region in *bou* rescued embryos and diffuses baso-laterally in mutants affecting the SJ organisation, such as in *mega bou* and *bou nrv2* double mutant backgrounds. Our observations reveal a third possibility, as a fraction of exogenous mCheBou is internalised in *bou* rescued embryos and, to a larger extent, in *bou Nrg* double mutants. In fact, in this latter genetic background virtually no mCheBou is observed in the cell cortex, indicating that the *Nrg* contribution is essential for Bou retention at the membrane level.

We show that mCheBou is internalised in vesicles containing at least one SJ constituent: the Kune protein. It is thus possible that Bou could be part of a complex that includes Kune, but most likely other components. In fact, although mCheBou does not accumulate at the membrane level in *bou kune* double mutant embryos, this protein is still internalised in this genetic context. Thus, other membrane components could be responsible for mCheBou capture in this background, suggesting that Kune may not be a Bou direct partner.

### A module containing Kune and Bou is internalised during epithelial morphogenesis

Previous results have shown that many SJ components are endocytosed during the early stages of SJ formation (stage 13 embryos) and subsequently re-exported to the membrane for their reallocation into the sub-apical region of ectodermal cells [44]. After this remodelling phase, a set of membrane proteins called SJ core components interact with each other and form a stable complex in the sub-apical membrane, with defects in SJ integrity resulting in their rapid baso-lateral diffusion [16,17]. Whether this lateral spreading contributes to the reduction of the Nrg-GFP or EGFPKune membrane signal observed in different SJ mutants is difficult to establish. However, our data show that some SJ constituents, such as Bou and Kune, are still endocytosed well after the initial remodelling phase, revealing a novel dynamic aspect of the SJ organisation.

In the case of Kune, our data reveal that a significant proportion of these molecules is internalised and degraded in embryonic epithelial cells at least until stage 16. However, a small pool of Kune molecules seems to reside in the baso-lateral membrane, as a weak mCheKune signal is observed in this location throughout development, both in wild type embryos and in absence of other SJ components. It is thus possible that Kune endocytosis could preferentially occur at the SJ level and proceed at a slower rate in other membrane subdomains. In contrast, we did not detect internal vesicles containing Nrg-GFP in wild type embryos or in mutant conditions, indicating that Nrg remains in the membrane after the SJ remodelling phase, as previously reported [44]. Our observations thus raise the possibility that stable structures integrating the SJ core components could interact at the membrane level with other modules exhibiting a high membrane turnover [17]. In addition, the fact that Kune internalisation is specifically enhanced in *Nrg* but not in *nrv2* mutants indicates that the different SJ core components are not functionally equivalent. Nrg could thus fulfil a specific role preventing the internalisation of a dynamic module containing Kune and Bou.

The observed membrane turnover of the claudin Kune mirrors the dynamic behaviour of its multiple vertebrate homologs in the context of tight junction maintenance. In fact, vertebrate claudins are actively endocytosed and subsequently targeted for membrane recycling or degradation, both during normal development and in pathological conditions [45]. As claudins are instrumental for regulating the selective permeability properties of vertebrate occluding junctions, our findings raise the possibility that their insect counterparts could also participate in dynamic processes affecting the properties of paracellular barriers. However, much remains to be learned about the actual roles of invertebrate claudins, as septate and tight junctions have very different structural organisations [20].

From a developmental perspective, endocytosis of specific SJ components could contribute to the plasticity of these adhesion structures and facilitate cell contact remodelling during epithelial morphogenesis. Consistently, Kune internalisation seems to operate according to a tissue-specific logic. In fact, in contrast with its dynamic localisation in other tissues, we have found that Kune is more stably associated to the SJ region in the embryonic salivary glands or in the larval subperineural glia. Whether the insect SJ contain other dynamic modules, including perhaps additional claudins and Ly6 proteins remains an open possibility. Future research aimed at understanding the organisational plasticity of these complex adhesion structures could reveal additional parallelisms with the vertebrate tight junctions and enhance our understanding of the mechanisms regulating their physiological and developmental properties.

## Materials and methods

### Drosophila strains

We used embryonic lethal null or strong loss of function alleles: *bou*^*6Ea2*^ [18], *kune*^*c309*^ [8], *Nrg*^*I7*^ [9], *mega*^*G0012*^ [6], *cold*^*f05607*^ and *crok*^*KG06053a*^ [23], *NrxIV*^*4304*^ [10], *Cont*^*ex956*^ [12], *sinu*^*nwu7*^ (Wu 2004), *nrv2*^*k13315*^ [11], *mmy*^*IK63*^ [46] and *mmy*^*GA74*^ [47]. We employed as cellular markers *Nrg-GFP* [25], α*Tub84B*::*YFP-Rab5* and *αTub84B*::*YFP-Rab7* [31] and *Lamp1-YFP*^*CPTI001775*^ [48]. The UAS constructs *UAS-GPI-GFP* [27], *UAS-CD63-GFP* [29], *UAS-Secreted-GFP* [26], *UAS-mCD8-GFP* and *UAS-mCD8mCherry* were expressed using the *HedgehogGAL4* driver. Balancers used include *FM7c twistGAL4 UAS-GFP*, *FM7c KruppelGAL4 UAS-GFP*, *CyO sChFP2*, *CyO twistGAL4 UAS-GFP*, *FM7c actinLacZ*, *CyO wgLacZ* and *TM6b iab-2LacZ*. All crosses were carried out at 25°C in standard cornmeal medium. Full definitions of all the stocks can be found in Flybase [49].

### Transmission electron microscopy

For ultra-structural analysis, we followed our previously published protocol [50]. In brief, stage 17 wild type and *bou* mutant embryos were immobilised by high-pressure freezing in a Bal-Tec HPM 010 (Balzers, Lichtenstein) high-pressure freezer. Embryos were subsequently fixed with 2% osmium tetroxide, 0.5% uranyl acetate and 0.5% glutaraldehyde in acetone at -90°C for 32 h, at -60°C and -40°C for 4 h at each step. The samples were washed with acetone before being transferred to an acetone-Epon mixture at -30°C (1:1 for 4 h, 1:2 for 12 h). They were warmed up to room temperature, infiltrated in Epon and polymerised at +60°C for 48 h. Ultrathin sections (about 70 nm) were contrasted with 2% uranyl acetate and 0.4% lead citrate. Sections were analysed in a Philips CM10 electron microscope at 60 kV.

### Constructs

The UASmCheBou construct was built cloning in frame the mCherry coding sequence into an NheI restriction site of the UAS-HABou plasmid, upstream of the Bou Ly6 domain [18]. For the *kune* constructs, a 4.3 kb fragment beginning at the *kune* start codon and containing the full coding sequence and most of its 3’ intergenic region was amplified by PCR from genomic DNA and cloned into the P(CaSpeR-4) vector [51]. The EGFP and the mCherry coding sequences and a fragment containing the 1.8 kb *kune* 5’ intergenic region were subsequently cloned in frame, upstream of the *kune* coding unit. All the constructs were sequenced and independent genomic insertions generated according to standard genetic procedures. For imaging of Kune, we selected two P(CaSpeR-4) insertions expressing robust levels of EGFPKune and mCheKune in both immunostained and live samples, but other insertions gave similar results. One copy of each selected insertion is capable of rescuing the SJ phenotypes observed in *kune* mutants (S3 Fig).

### Live imaging and quantification

Embryos from overnight collections were dechorionated in bleach, rinsed in water and mounted *en masse* with a paintbrush on a gas permeable membrane fixed over a window made in a cardboard slide. Embryos were then imbibed in a thin layer of Halocarbon 700 oil (Sigma) and covered with a movable coverslip resting on two lateral spacers. With this method, live embryos can be staged and genotyped under the microscope and reoriented for imaging of their ventral epidermis. All images shown correspond to single optical sections. The planar views of the epidermis correspond to a sub-apical focal plane, allowing simultaneous visualisation of the SJ region and the different pools of internal vesicles. Photo-bleaching of the mCheBou signal was conducted on a Zeiss LSM710 confocal microscope equipped with a 40×/1.3 Oil DIC M27 objective, passing 4 times over 9 z positions with the 561nm laser at 80% and a 50.42 μs pixel dwell time. Live explants from third larval instar larval tissues were dissected and mounted on a microscope slide in Schneider S2 culture cell medium (Gibco) and subsequently enclosed with a coverslip resting over a double-sided tape spacer. Samples were imaged within 30 minutes after dissection. Confocal images were obtained on Leica SP2, Leica SP8 and Zeiss LSM710 confocal microscopes and processed using Image-J and Photoshop.

For vesicle quantification, we counted for each genotype the total number of mCheBou, YFP-Rab7 and double positive vesicles present in randomly chosen square fields of 100 μm^2^, covering the apical surface of the ventral epidermis of live stage 16 embryos. Average number of vesicles and standard deviations were calculated pooling the results of at least 13 different fields for each genotype. For quantification of the EGFPKune and mCheKune signal at the cell junctions, we scored the maximal fluorescence intensity observed in linear plots drawn perpendicular to the membrane, using the Image-J Plot-Profile tool (S5 Fig). Values for each genotype were obtained for at least 48 different junctions imaged in confocal sections corresponding to 8–10 different embryos.

### Immunostaining procedures

Embryos and larval tissues were fixed for 20 minutes in 4% paraformaldehyde in PBS. Subsequent blocking, washings and over night incubation with primary and secondary antibodies were carried out in 0.1% Triton-X100, 0.1% BSA. Antibodies used include rabbit anti-Kune 1/100 [8], rat 5F8 anti-RFP 1/400 (Chromotek), rabbit anti-GFP 1/500 (Torrey), rabbit anti-βGal 1/1000 (Cappel) and the mouse monoclonal 7G10 anti-FasIII 1/30 (DSHB), C566.9 and C615.16 anti-Coracle 1/100 (DSHB). Anti-rabbit FITC 1/400, anti-mouse TRITC 1/400 (Jackson labs) and anti-rat Alexa555 1/500 (Invitrogen) were used as secondary antibodies. Samples were mounted in Vectashield (Vector).

### Xbp1 reverse transcription and PCR

For each genotype, RNA was extracted from 40 stage 16–17 embryos using the RNeasy extraction kit (Qiagen), adding an on-column DNase digestion step. For each RT reaction, 1 μg of total RNA was reverse transcribed using Superscript II (Invitrogen) and the *Xbp1* specific primer CCGAGTGTAGAGACAATGCG, according to the manufacturer's instructions. PCR was run for 25 cycles, as in [33], adding 3 μl of the corresponding RT reactions as a matrix. PCR products were analysed in a 3.5% low melting agarose gel in TBE buffer.

## Supporting information

S1 Fig mCheBou co-localises with Nrg-GFP in the epidermis throughout development. (A-E) Confocal images of the ventral epidermis of live *bou* rescued embryos expressing Nrg-GFP (green) and mCheBou (magenta and b/w, lower panels), taken at different developmental stages using the same acquisition settings. The mCheBou protein is produced in the *HhGAL4* domain and is incorporated by epidermal cells at the membrane level, where it co-localises with Nrg-GFP. The levels of both proteins increase in parallel throughout embryonic development. (F-G’) Confocal images showing a transversal section of the epidermis in *Nrg-GFP UASmCheBou* stage 16 embryos. In presence of the *HhGAL4* driver, a diffuse extracellular signal (magenta in F, b/w in F’, labelled with asterisks) is detected between the vitelline membrane (red arrowheads) and the epidermis, marked with Nrg-GFP (shown in green, white arrows). This perivitelline signal is totally absent in control embryos lacking the driver (G,G’). Scale bars: 15 μm.
(TIF)

S2 Fig Kune localisation in mutants for different SJ components. (A) Confocal images showing the lateral epidermis of stage 16 embryos immunostained for Kune (green in left panels, b/w in right panels) and Coracle (red, left panels). Genotypes are indicated above each panel. Kune staining is not detected in *kune* embryos but is visible in the cell membrane of wild type, *sinu*, *crok*, *NrxIV*, and *Cont* mutants. Scale bar: 10 μm. (B) Agarose gel showing *Xbp1* RT-PCR products recovered from wild type (*WT*) and mutant embryos of different genotypes, as indicated above each lane. The 127 bp and 104 bp bands correspond respectively to unspliced and spliced *Xbp1* transcript forms. The lower band is overrepresented in ER stress conditions, as in *mmy* mutant embryos. Wild type, *bou*, *Nrg* and *mega* embryos display indistinguishable *Xbp1* splicing patterns. A control PCR reaction was loaded using as a matrix a RT reaction where no reverse transcriptase was added (*WT*, no RT lane).
(TIF)

S3 Fig Constructs engineered for expression of Kune tagged forms and rescue experiments. (A) Diagram showing the *kune* region and the genomic fragment incorporated into the EGFPKune and mCheKune constructs. The two fluorescent tags were added to the N-terminus of the protein. (B-G) Confocal images of stage 16 *kune* mutant embryos immunostained with a FasIII antibody, revealing the SJ organisation in the hindgut (B-D) and the salivary glands (E-G). FasIII mislocalises over the lateral membrane in *kune* mutant embryos (B,E, red arrows). This phenotype is rescued in presence of a copy of EGFPKune (C,F) or mCheKune (D,G). Scale bar: 16 μm.
(TIF)

S4 Fig Live imaging reveals Kune membrane turnover in the embryonic epidermis. Confocal optical sections showing the localisation of EGFPKune and mCheKune in the ventral epidermis of wild type embryos of different developmental stages. For each stage, the left panels (Antibody) correspond to samples immunostained with specific antibodies against GFP (green or b/w in middle panels) and mCherry (red or b/w in lower panels). The right panels (Live) present the EGFPKune and mCheKune fluorescent signals directly observed in live samples. An EGFPKune membrane signal is already detectable at stage 11 and its levels increase gradually over time in live embryos. The mCheKune membrane signal becomes apparent by stage 12 and is seen at low levels in this location until stage 17. Immunostained samples reveal that EGFPKune and mCheKune co-localise at the cell membrane. Scale bar: 10 μm.
(TIF)

S5 Fig Quantification of the EGFPKune and mCheKune fluorescent signals in cell junctions. (A) Each measure (for each genotype, n≥48) corresponds to the fluorescent intensity of EGFPkune (green) or mCheKune (red) detected at a randomly chosen epidermal cell junction in stage 16 live embryos. Mean values obtained are indicated by black bars. The EGFPKune signal levels drop in *mega*, *bou* and *Nrg* mutants (p<0.0001, Student’s t-test), whereas the mCheKune values remain comparable in all the backgrounds analysed. (B) Each measure corresponds to the maximal florescence intensity observed in a linear plot drawn perpendicularly to the cell junction.
(TIF)
