# Supplementary figures and images for: Boudin trafficking reveals the dynamic internalisation of specific septate junction components in Drosophila
Source: PLoS One. 2017 Oct 4;12(10):e0185897. doi: 10.1371/journal.pone.0185897 (PMC5627947; doi:10.1371/journal.pone.0185897)

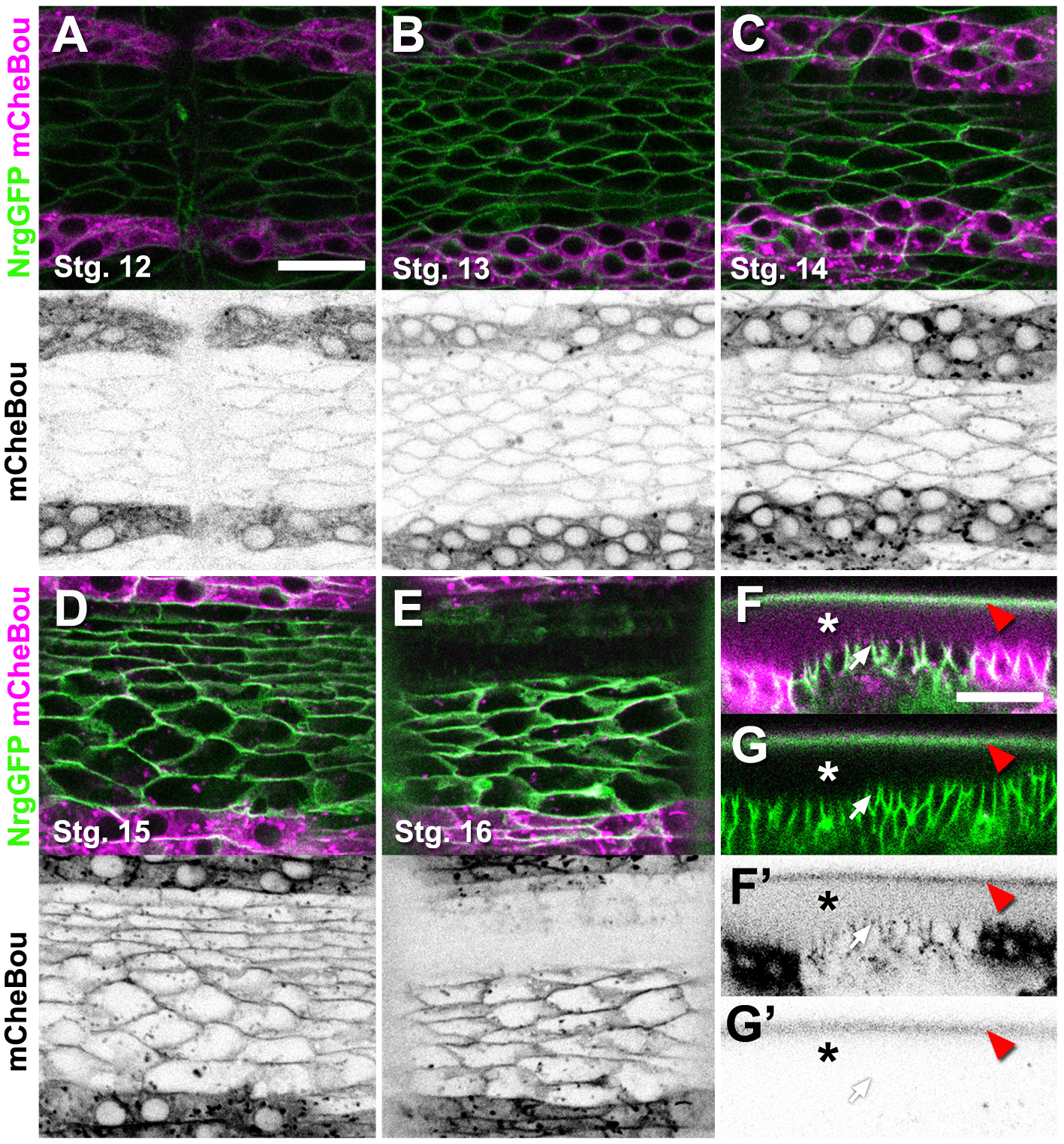

Supplement: S1 Fig — (A-E) Confocal images of the ventral epidermis of live bou rescued embryos expressing Nrg-GFP (green) and mCheBou (magenta and b/w, lower panels), taken at different developmental stages using the same acquisition settings. The mCheBou protein is produced in the HhGAL4 domain and is incorporated by epidermal cells at the membrane level, where it co-localises with Nrg-GFP. The levels of both proteins increase in parallel throughout embryonic development. (F-G’) Confocal images showing a transversal section of the epidermis in Nrg-GFP UASmCheBou stage 16 embryos. In presence of the HhGAL4 driver, a diffuse extracellular signal (magenta in F, b/w in F’, labelled with asterisks) is detected between the vitelline membrane (red arrowheads) and the epidermis, marked with Nrg-GFP (shown in green, white arrows). This perivitelline signal is totally absent in control embryos lacking the driver (G,G’). Scale bars: 15 μm. (TIF) [file pone.0185897.s001.tif]

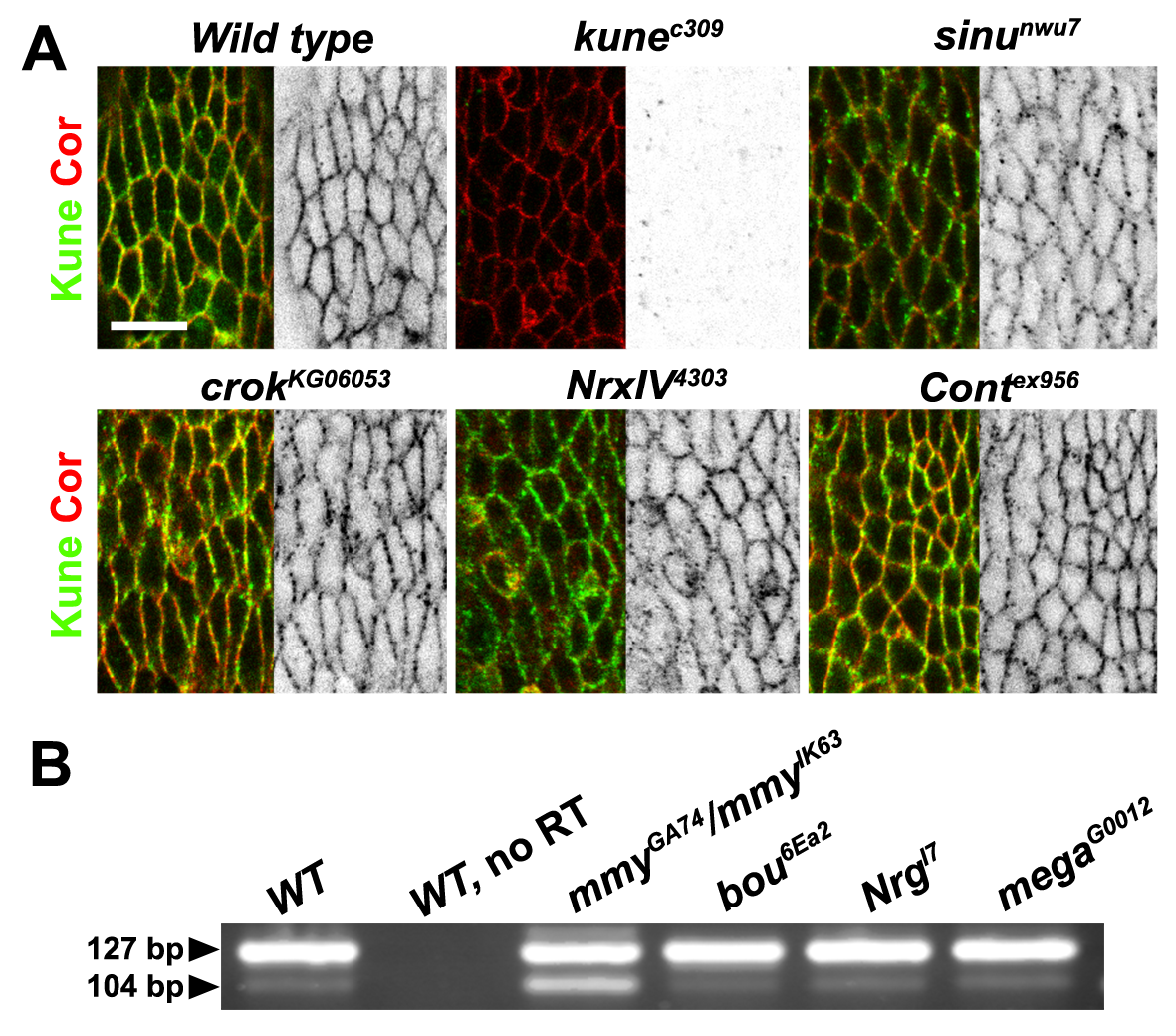

Supplement: S2 Fig — (A) Confocal images showing the lateral epidermis of stage 16 embryos immunostained for Kune (green in left panels, b/w in right panels) and Coracle (red, left panels). Genotypes are indicated above each panel. Kune staining is not detected in kune embryos but is visible in the cell membrane of wild type, sinu, crok, NrxIV, and Cont mutants. Scale bar: 10 μm. (B) Agarose gel showing Xbp1 RT-PCR products recovered from wild type (WT) and mutant embryos of different genotypes, as indicated above each lane. The 127 bp and 104 bp bands correspond respectively to unspliced and spliced Xbp1 transcript forms. The lower band is overrepresented in ER stress conditions, as in mmy mutant embryos. Wild type, bou, Nrg and mega embryos display indistinguishable Xbp1 splicing patterns. A control PCR reaction was loaded using as a matrix a RT reaction where no reverse transcriptase was added (WT, no RT lane). (TIF) [file pone.0185897.s002.tif]

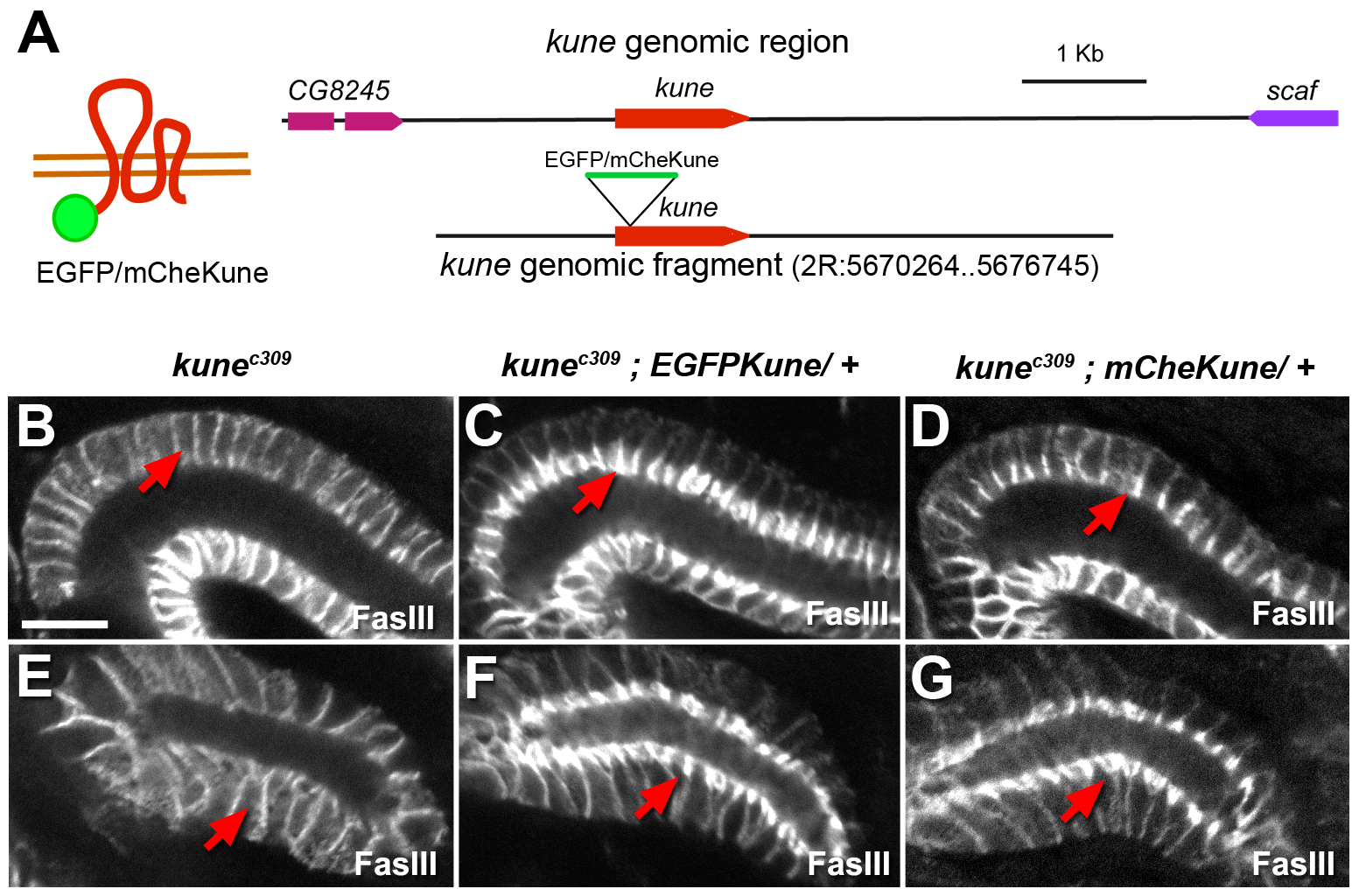

Supplement: S3 Fig — (A) Diagram showing the kune region and the genomic fragment incorporated into the EGFPKune and mCheKune constructs. The two fluorescent tags were added to the N-terminus of the protein. (B-G) Confocal images of stage 16 kune mutant embryos immunostained with a FasIII antibody, revealing the SJ organisation in the hindgut (B-D) and the salivary glands (E-G). FasIII mislocalises over the lateral membrane in kune mutant embryos (B,E, red arrows). This phenotype is rescued in presence of a copy of EGFPKune (C,F) or mCheKune (D,G). Scale bar: 16 μm. (TIF) [file pone.0185897.s003.tif]

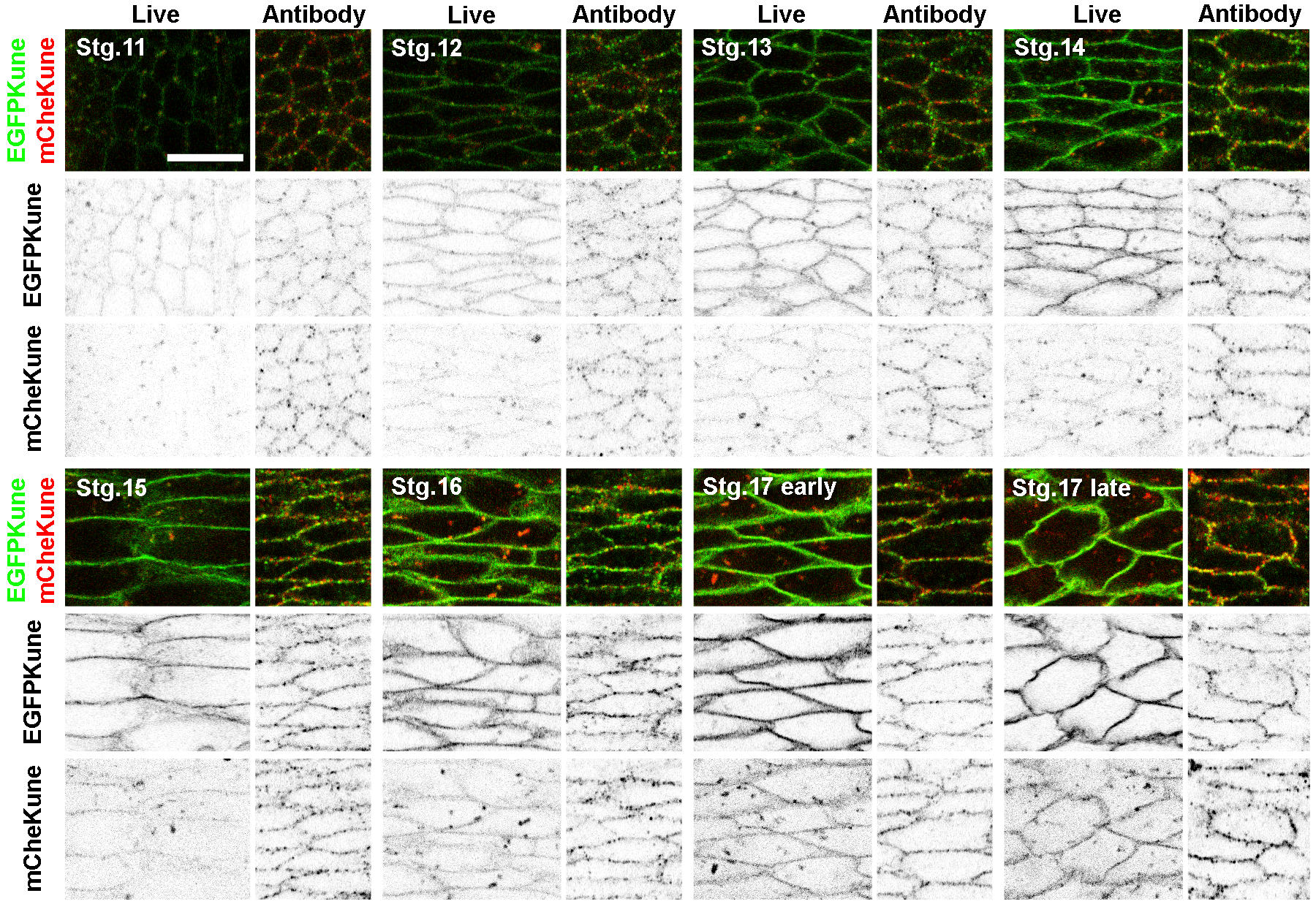

Supplement: S4 Fig — Confocal optical sections showing the localisation of EGFPKune and mCheKune in the ventral epidermis of wild type embryos of different developmental stages. For each stage, the left panels (Antibody) correspond to samples immunostained with specific antibodies against GFP (green or b/w in middle panels) and mCherry (red or b/w in lower panels). The right panels (Live) present the EGFPKune and mCheKune fluorescent signals directly observed in live samples. An EGFPKune membrane signal is already detectable at stage 11 and its levels increase gradually over time in live embryos. The mCheKune membrane signal becomes apparent by stage 12 and is seen at low levels in this location until stage 17. Immunostained samples reveal that EGFPKune and mCheKune co-localise at the cell membrane. Scale bar: 10 μm. (TIF) [file pone.0185897.s004.tif]

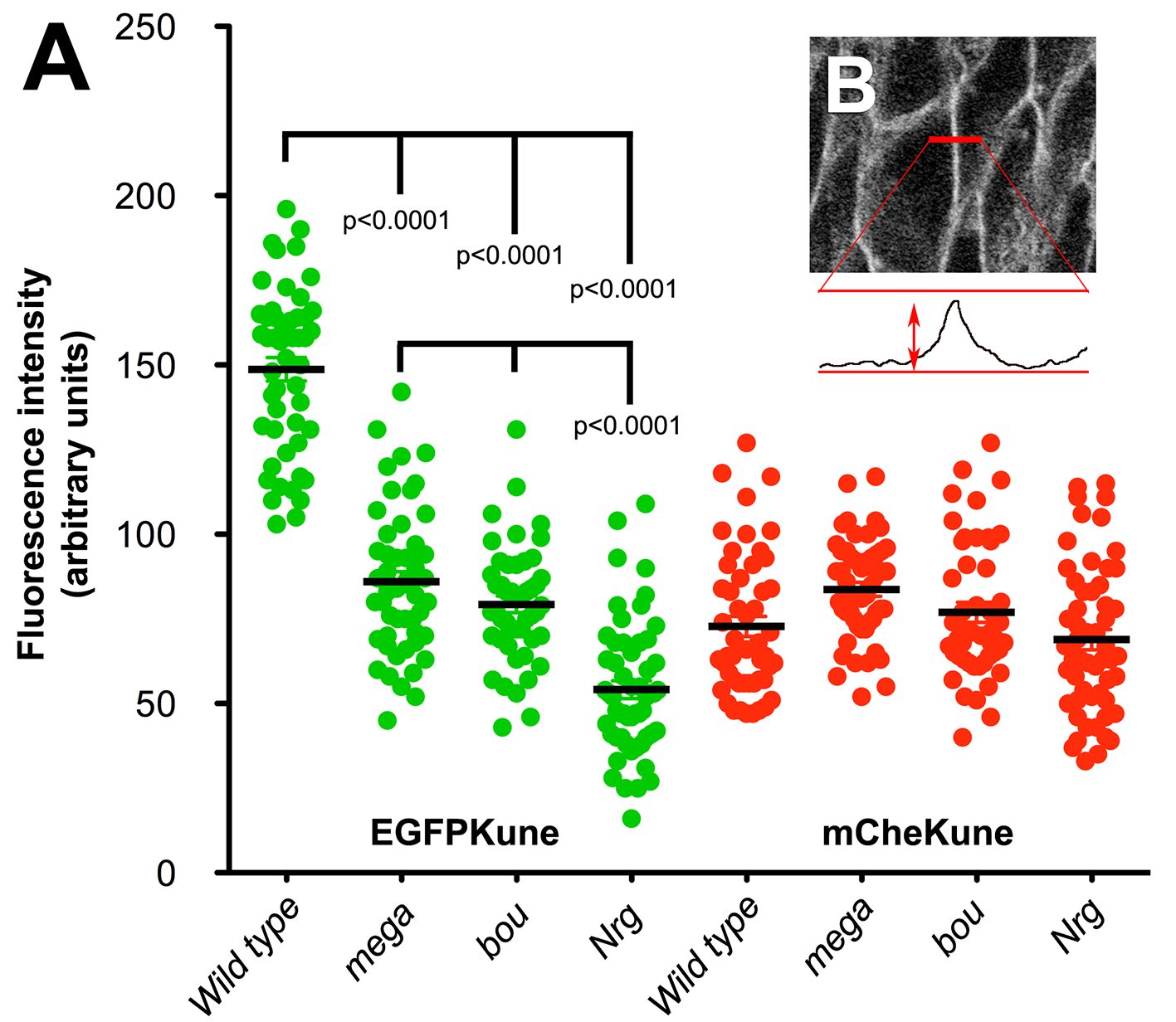

Supplement: S5 Fig — (A) Each measure (for each genotype, n≥48) corresponds to the fluorescent intensity of EGFPkune (green) or mCheKune (red) detected at a randomly chosen epidermal cell junction in stage 16 live embryos. Mean values obtained are indicated by black bars. The EGFPKune signal levels drop in mega, bou and Nrg mutants (p<0.0001, Student’s t-test), whereas the mCheKune values remain comparable in all the backgrounds analysed. (B) Each measure corresponds to the maximal florescence intensity observed in a linear plot drawn perpendicularly to the cell junction. (TIF) [file pone.0185897.s005.tif]
